# Training Programmes Can Change Behaviour and Encourage the Cultivation of Over-Harvested Plant Species

**DOI:** 10.1371/journal.pone.0033012

**Published:** 2012-03-14

**Authors:** Sophie J. Williams, Julia P. G. Jones, Colin Clubbe, James M. Gibbons

**Affiliations:** 1 School of Environment, Natural Resources and Geography, Bangor University, Gwynedd, United Kingdom; 2 Conservation, Living Collections and Estates Directorate, Royal Botanic Gardens, Kew, Richmond, Surrey, United Kingdom; Madagascar Fauna Group, United States of America

## Abstract

Cultivation of wild-harvested plant species has been proposed as a way of reducing over-exploitation of wild populations but lack of technical knowledge is thought to be a barrier preventing people from cultivating a new species. Training programmes are therefore used to increase technical knowledge to encourage people to adopt cultivation. We assessed the impact of a training programme aiming to encourage cultivation of xaté (*Chamaedorea ernesti-augusti)*, an over-harvested palm from Central America. Five years after the training programme ended, we surveyed untrained and trained individuals focusing on four potential predictors of behaviour: technical knowledge, attitudes (what individuals think about a behaviour), subjective norms (what individuals perceive others to think of a behaviour) and perceived behavioural control (self assessment of whether individuals can enact the behaviour successfully). Whilst accounting for socioeconomic variables, we investigate the influence of training upon these behavioural predictors and examine the factors that determine whether people adopt cultivation of a novel species. Those who had been trained had higher levels of technical knowledge about xaté cultivation and higher belief in their ability to cultivate it while training was not associated with differences in attitudes or subjective norms. Technical knowledge and perceived behavioural control (along with socio-economic variables such as forest ownership and age) were predictors of whether individuals cultivate xaté. We suggest that training programmes can have a long lasting effect on individuals and can change behaviour. However, in many situations other barriers to cultivation, such as access to seeds or appropriate markets, will need to be addressed.

## Introduction

Humans have carried out wild harvesting of plant species for subsistence and trade for thousands of years [1]. However, over-exploitation now threatens many wild plant populations [2]. There has been increasing interest in the cultivation of harvested plant species as a method to reduce over-exploitation of wild populations [3]–[4] and also to improve human livelihoods [5]. The assumption is that increasing domestic supply will reduce the pressure on wild populations [6]–[8]. It is likely that multiple factors determine an individual's decision to begin cultivation, including socio-economic characteristics [9], land tenure [10], risk preference [11] and technical knowledge about cultivating a novel species [12]. Training programmes have been initiated to encourage cultivation of over-harvested species [13]–[14]. Such programmes implicitly assume that lack of technical knowledge is the barrier to cultivation. However few studies have explicitly considered individuals' decision-making processes concerning whether to engage in cultivation or not, and how this may be influenced by a training programme [15]–[16].

**Figure 1:**
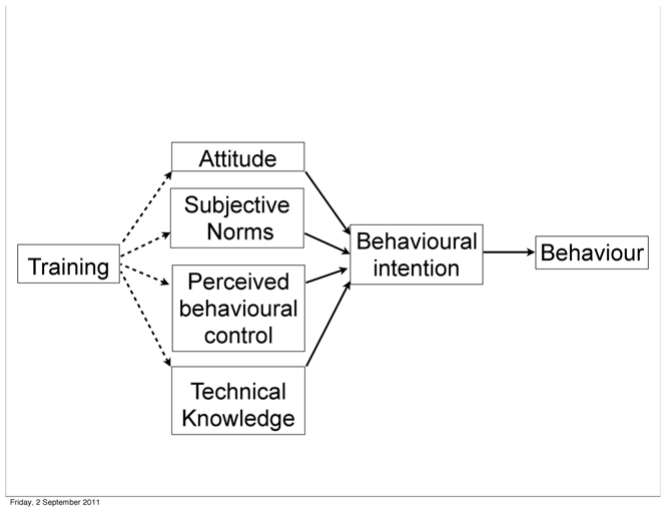
The theory of planned behaviour [**18**]. In this conceptual model we include the additional variable of technical knowledge as a predictor of behavioural intention and training as a potential method of influencing the four predictors of behavioural intention.

Understanding the drivers of human decision making and behaviour is important for improving the design of effective conservation interventions [17]. The theory of planned behaviour [18] provides a useful framework to analyse individual behaviour. This social psychological theory uses three factors: attitudes, subjective norms and perceived behavioural control, as predictors of behavioural intention, the antecedent to behaviour (figure 1). Attitudes can be conceptualised as what an individual thinks about a behaviour and can be favourable or unfavourable. Subjective norms describe what individuals perceive others to think of a behaviour [18]. Perceived behavioural control (PBC) is a self-assessment of whether a behaviour can be enacted successfully and also the availability of the resources to perform the behaviour [19]. Some studies suggest knowledge is also an important predictor of behaviour, however a personal assessment of knowledge may not necessarily reflect the accuracy of the knowledge [20]. Technical knowledge can be described as factual, accurate information about a specific behaviour [21] and can be included as a predictor of behaviour [21]–[23]. Recent work has examined the relative predictive power of technical knowledge on behavioural decisions compared to the other factors traditionally included in the theory of planned behaviour [20].

Training programmes aiming to initiate cultivation of a new plant species can address a perceived lack of technical knowledge in cultivation methods, and may also influence attitudes, subjective norms [10] and perceived behavioural control [24]. Previous research evaluating changes in behaviour frequently rely on self-reports of behavioural intentions; actual behaviour is often difficult to measure [25]–[27]. In this study we assess the impact of a training programme that aimed to promote the cultivation of an over-harvested palm species (Xaté - *Chamaedorea ernesti-augusti* H.A. Wendl.) among forest-edge communities in Belize. The leaves of xaté are used in the floricultural industry in a global trade worth approximately US $4 million annually [28]. This case study offers an excellent opportunity to investigate the relative importance of the various predictors of behaviour on actual behaviour as whether a participant went on to cultivate xaté is easily documented and readily verifiable. We use a modified theory of planned behaviour as a framework and combine qualitative and quantitative research methods to address 1) the impact of training on the participant's attitudes, subjective norms, perceived behavioural control and technical knowledge (whilst accounting for socioeconomic variables), 2) whether attitudes, subjective norms, perceived behavioural control and technical knowledge predict xaté cultivation behaviour (again controlling for socioeconomic variables), and 3) other barriers to xaté cultivation in Belize.

## Methods

### Study site

**Figure 2:**
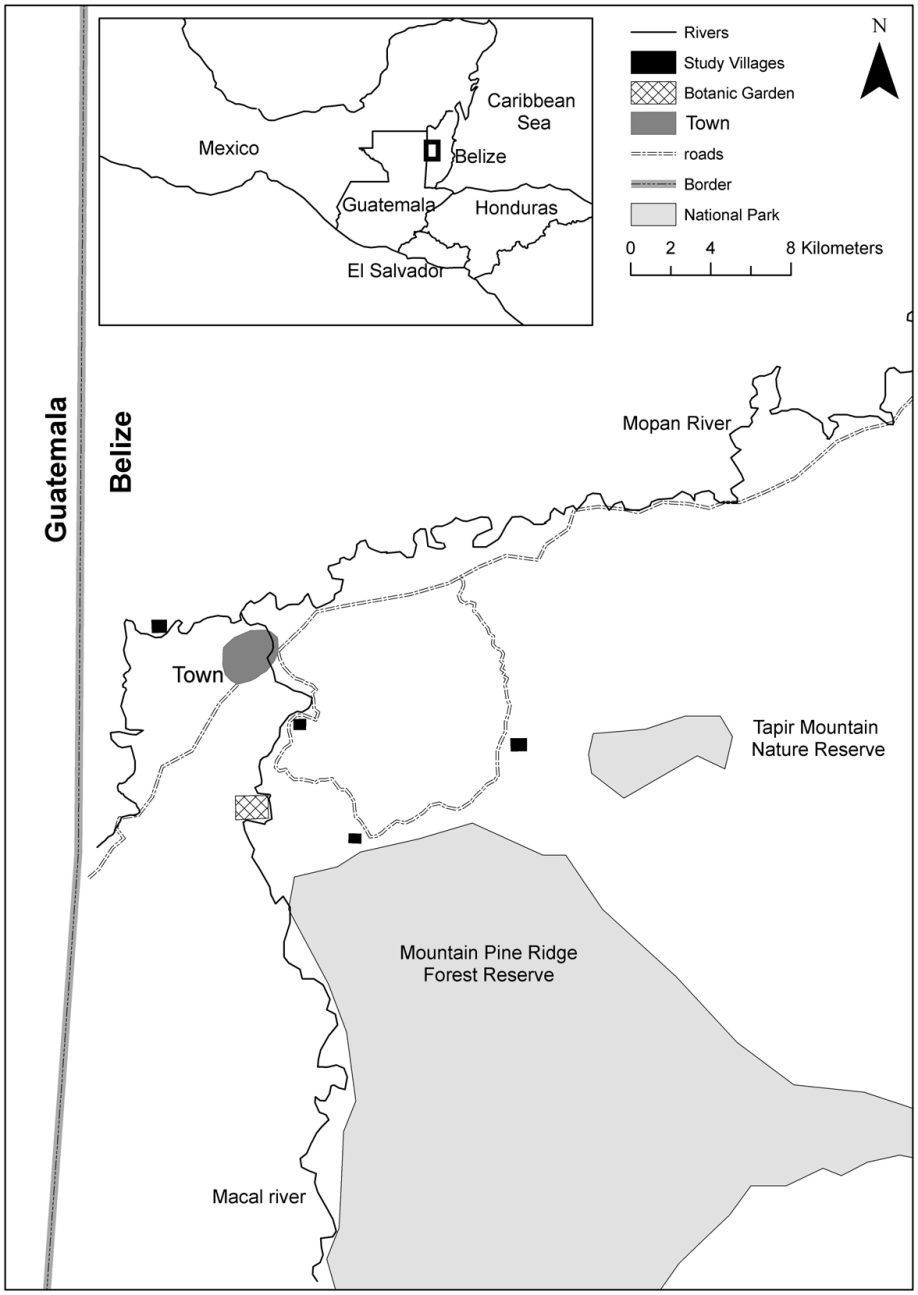
The location of study villages in Belize.

Belize is a small country on the Caribbean coast of Central America with a population of approximately 300,000 [29]. This work was carried out in the district of Cayo from December 2010 to February 2011 (figure 2). Small scale farming is the main occupation for the majority of the villagers and the inhabitants primarily speak Yucatec Mayan, but most people are also fluent in English and Spanish [29]. As part of a Darwin Initiative Project (UK government funding), Belize Botanic Garden prepared a xaté cultivation training programme which was delivered to 50 farmers from four villages in 2005 and provided participants with xaté seedlings to encourage cultivation. The botanic garden also planted a demonstration plot to promote xaté cultivation. Our study was carried out in these four villages (not named to preserve respondents' anonymity). The training programme aimed to teach people in Belize how to cultivate the xaté, as a method of increasing the supply from cultivated sources and improving local farmers' livelihoods. Creating a xaté market is a relatively new initiative in Belize, whereas Guatemala has a long established system and infrastructure for sorting, packing and exporting xaté leaf [28]. Wild harvesting of xaté is uncommon among Belizeans, and it is suggested that wild harvesting in Belize is carried out illegally by Guatemalans crossing the border [28].

### Questionnaire development and design

Belize Botanic Garden provided the training participants with information about xaté cultivation; we used this to develop our questions assessing technical knowledge (table 1). Our measure of technical knowledge is distinct from the assessment of the attitudes, subjective norms and perceived behavioural control as we are specifically examining the amount of accurate information an individual has, whereas the three other behavioural predictors do not measure the amount or accuracy of information held [20]. To develop the statements measuring attitudes, subjective norms and perceived behavioural control we held discussions with key informants to help us to understand the range of perceptions within the communities about xaté cultivation (table 2). The questionnaire was adapted following discussion with two key informants to reflect local context. Responses were initially measured on a five-point Likert scale. A pilot study (n = 10) found that people either agreed or disagreed with statements and it was difficult to elicit variation in the strength of opinion. For this reason, we simplified the survey to use a three-point scale. In the pilot study we constructed the individual attitude, subjective norms and perceived behavioural control statements to include target, action, context and timeframe [30]. For example, we could ask an individual their attitude towards cultivating xaté on a farm in the next five years. In this example cultivation is the action, xaté is the target, the farm is the context and five years is the timeframe. However, the detail in these statements was confusing for respondents. As the context and timeframe remained the same for each statement, we outlined these two components at the beginning of the attitude statement section of the questionnaire. The target and the action were defined individually for each statement. After these revisions, we carried out a second pilot study (n = 10) and no further changes were made and so these data were included in the final analysis. Respondents were also asked their age, length of time living in the village, amount of forest land owned, number of financial dependents, number of children, years of schooling and the length of time they had been a farmer. Following the questionnaire we carried out semi-structured interviews to gain a more nuanced understanding of farmers' perspectives of xaté cultivation. The following topics were discussed with informants: what are the barriers to cultivation of xaté? Is xaté cultivation a good investment of land and effort? Why don't Belizeans wild harvest xaté?

**Table 1:** Statements used to measure technical knowledge with answers based on information provided during training at Belize Botanic Garden.

Question	Correct answer	Rationale
What is xaté used for in the US?	Decoration, ornament, flower arranging	The only known use of the plant
How long xaté seeds take to germinate?	Between 9–12 months	Based on tests at the Belize Botanic Garden
What colour xaté seeds are when ready to harvest?	Black/purple	Distinct colour change from green when seeds are ripe
Have there been any changes in the numbers of xaté in the forests of Belize in the last ten years?	Yes, decline	A decline in xaté in Belize has been documented in the DI^1^ project
Does xaté require full sun or shade to grow?	Shade	Xaté is not tolerant of direct sun
How many xaté leaves you can take each year without harming the plant?	2 or <2	Based on research during DI project. Training programme taught 2 leaves per year maximum to be harvested.

1 DI = Darwin Initiative project 12012.

**Table 2:** Statements used to measure attitudes, subjective norms and perceived behavioural control, including a summary of responses from trained and untrained participants.

Predictor	Measurement^1,2^	Trained participants response^3^	Untrained participants response^3^
Attitude 1	Growing xaté is good way for farmers to earn money	8.6	8.6
Attitude 2	Growing xaté isn't a worthwhile use of land	7.6	7.2
Attitude 3	It is very difficult to earn money from growing xaté	6.3	6.7
Attitude 4	The risk of theft in this area is too high to make growing xaté worthwhile	9.6	7.8
Subjective Norms 1	My friends think it is a bad idea to grow xaté	7.4	7
Subjective Norms 2	It is important to grow the same crops as my friends	7.5	8
Perceived behavioural control 1	I know how to grow xaté	4.7	2.8
Perceived behavioural control 2	I don't have the money to buy what I need to grow xaté	9.2	4.5

1 Informants were told each statement was based in the local area, in the next five years, to include target, action, context and timeframe in the statements.

2 Statements were coded so positive attitudes, subjective norms and PBC likely to favour xaté cultivation had higher values.

3 Values are rescaled to range between 0–10; all items were re-coded so high values indicated a positive view of xaté cultivation.

### Sampling strategy and data collection

The xaté cultivation training coordinated by Belize Botanic Garden targeted farmers: i.e. those people dependent on cultivating crops for their primary source of income. We aimed to contact all participants of the training programme through three key informants (Curator of Belize Botanic Garden, Chairman of the local Farmers Association and a local agroforestry non-government organisation (NGO) representative). From the total of 50 people trained in xaté cultivation, we were able to interview 38. The people not interviewed had either moved away from the village or were not available for interview. To provide a random sample of the farmers who were not involved with the training programme, we compiled a comprehensive list of all the farmers in the villages through discussions with three key informants independently (Chairman of the local Farmers Association, the agroforestry NGO representative, a farmer that had lived and farmed in the village for 45 years). We assume that this list of 122 farmers is reliable as there was excellent agreement in the names provided by the three informants (117 names were the same on each informant's list with an extra 4 or 5 provided by two informants). From this list, 50 farmers were randomly sampled using numbers generated at random by R [31]. All farmers interviewed, except two, were male. Interviews were arranged by visiting the house of the farmer and organising a time that would be suitable to conduct the survey. Interviews were carried out in English by SW and a British research assistant, at the houses of farmers or on their farmland, whichever was more convenient for the informant.

An ethics checklist, as required by Bangor University, was completed prior to data collection and indicated that the research did not require further review (see Supplementary Material S1 for ethics statement). Oral consent was obtained from all study informants and all data were stored anonymously.

### Data Analysis

The quantitative data measuring attitudes was assessed for internal consistency using the Cronbach's Alpha [32]. The four items measuring attitude showed moderate internal consistency at the level 0.68 and so could be used as a single measure of attitude. Items were coded so positive answers towards cultivating xaté had higher scores. The scores for attitude, subjective norm and perceived behavioural control were a simple calculation in accordance with Ajzen (2006) [33]. To provide a single attitude measure the four statement scores were summed. Scores of the two items measuring subjective norms were summed, as were the scores of the PBC statements. In a Theory of Planned Behaviour framework, where possible, evaluation statements should correspond directly to belief statements [18]. In this case indexes can be constructed by multiplying the scores together [33]. In this study a direct link was not possible for statements so we use a simpler approach of adding scores together. This alternative construction did not substantially affect the results. Xaté technical knowledge scores were calculated by adding all the correct answers for the technical knowledge questions to provide a measure between 0 and 6. These four variables (attitude, subjective norms, PBC and technical knowledge) were then rescaled to a common range between 0 and 40 to allow direct comparisons. The higher scores represent positive attitudes, social norms, PBC and higher technical knowledge. Socio-demographic variables were re-scaled to allow direct comparison between all variables.

Demographic and socio economic characteristics of the trained and untrained informants were compared using a Student's t test. We then used proportional odds logistic models [34] and [35] to assess the impact of training on the participants' attitudes, subjective norms, perceived behavioural control and technical knowledge. This method allows for ordinal response variables to be fitted in the model. We controlled for socio-demographics by including the amount of forest owned, years at school and age in the models. For each response variable we developed a candidate set of 8 models, which were ranked by Akaike information criterion (AIC), a method used to measure the goodness of fit of a model. We use the corrected AIC (AICc) to account for our small sample size. For each candidate set there was no single model with clear support so we used model averaging to estimate the parameter coefficients. Uncertainty in parameter estimates was calculated as according to Burnham and Anderson 2002 [36].

To assess whether attitudes, subjective norms, perceived behavioural control and technical knowledge predict behaviour we used a generalised linear model with xaté cultivation as the binomial response variable and a logit link function. We developed a candidate set of 24 models *a priori* and included the following predictor variables: age, years at school, amount of forest owned, attitude, subjective norms, perceived behavioural control and knowledge (table 3). The quadratic functions of age and school were included to allow for a potential non-linear response to these variables. Because of the limited sample size (n = 87), interactions between variables were not included in the models. The AICc was used to rank the candidate models and to calculate the relative weight of each model. The predicted probability of xaté cultivation was estimated under scenarios of varying technical knowledge and perceived behavioural control. Uncertainty in parameter values was incorporated by drawing 1000 times from a multivariate normal distribution with coefficient mean and covariance estimates from the best model [37]. Other variables were set at the sample medians.

**Table 3:** Summary of 10 candidate models with lowest AICc developed to assess the predictors of xaté cultivation.

model	forest	age	age^2^	school	school^2^	know	attitude	SN*	PBC*	AICc*	ΔAICc	Weight
1	√	√				√			√	67.75	0.00	0.21
2	√	√				√		√	√	68.21	0.46	0.17
3	√	√	√			√	√	√	√	68.32	0.57	0.16
4	√	√		√	√	√	√	√	√	69.76	2.01	0.08
5	√	√	√	√	√	√	√		√	69.98	2.23	0.07
6	√	√				√	√	√	√	70.18	2.43	0.06
7	√	√	√	√		√	√	√	√	70.70	2.96	0.05
8		√	√	√	√	√	√	√	√	71.29	3.55	0.04
9	√	√		√		√	√	√	√	71.29	3.55	0.04
10	√	√				√	√			71.83	4.09	0.03

* SN: social norm, PBC: perceived behavioural control, AICc: corrected Akaike Information Criterion.

To analyse the qualitative data, key statements relevant to the topics outlined for the semi-structured interviews were extracted from the audio files and transcribed.

## Results

### Summary of the sample

The mean duration of the interviews was 24 minutes, with a maximum of 55 minutes. A total of 87 people were interviewed (trained = 38, untrained = 49). No significant differences in socio economic variables between the trained and untrained informants were found (p<0.05, df = 86). Twenty-six people were actively cultivating xaté (trained = 22, untrained = 4). Mean scores for the four attitude and two subjective norm statements indicate the majority of farmers have positive attitudes and subjective norms towards xaté cultivation, irrespective of training (table 2). The responses for perceived behavioural control indicates that untrained participants have less confidence in their abilities and less access to resources for cultivation (table 2).

### What does training influence?

**Figure 3:**
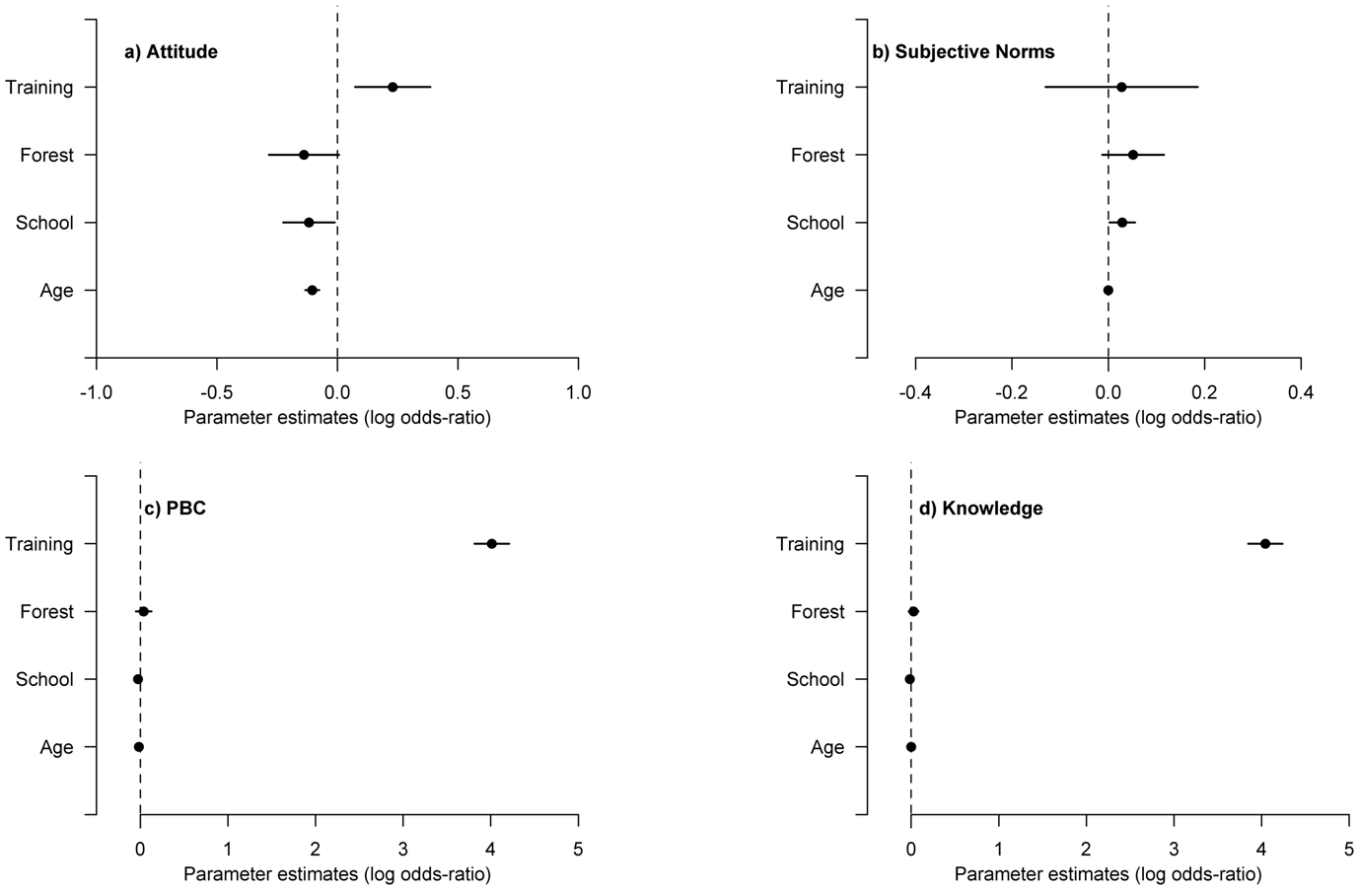
Model averaged parameter estimates. Illustrating the influence of training upon attitudes (a), subjective norms (b), perceived behavioural control (c) and technical knowledge (d), controlling for the socio-demographic variables of forest ownership, years at school and age. 3a and 3b indicate training has had little impact on attitudes and subjective norms whereas figures 3c and 3d show a positive impact of training on perceived behavioural control and technical knowledge. The central circles are the mean coefficient estimate for each parameter. Lines indicate 95% confidence intervals. Socio-demographic variables were rescaled to allow direct comparison with the training variable.

Whilst controlling for socio-demographic variables, we found training had a small positive impact on attitudes (figure 3a) and no evidence of influence upon subjective norms (figure 3b). Training influenced perceived behavioural control (self belief in their abilities to cultivate xaté and also access to resources needed to cultivate - figure 3c) and technical knowledge i.e. the amount of accurate information an individual has about cultivation of xaté (figure 3d).

### What predicts cultivation?

**Figure 4:**
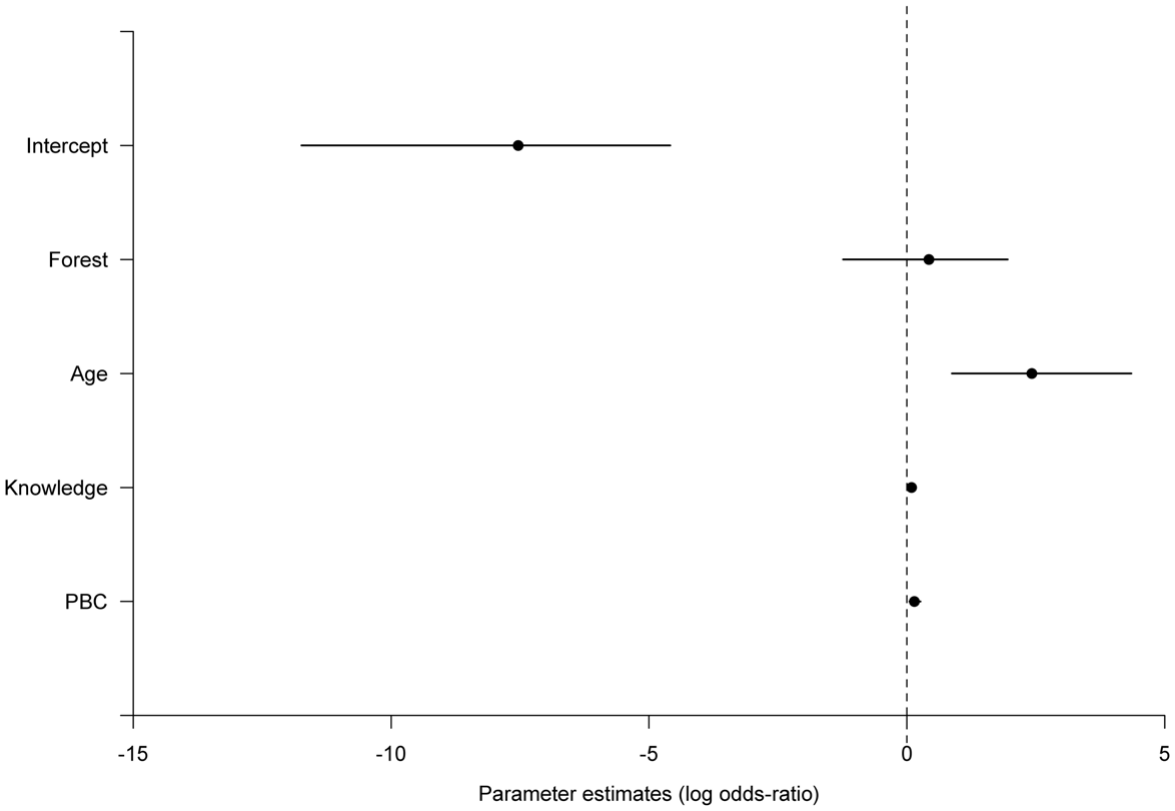
Coefficient plot showing the estimates for model averaged coefficients predicting xaté cultivation. The central circles are the mean coefficient estimate for each parameter and lines indicate 95% confidence intervals. There is a positive effect of age, PBC and knowledge on cultivation while there is no significant effect of forest ownership. In the absence of these factors the low value of the intercept suggests that little cultivation would occur.

Table 3 presents a summary of 10 (of the 24) candidate models with the lowest AICc, developed to predict xaté cultivation. Three models are >2 ΔAICc but as the model with the lowest AICc also has the lowest number of parameters, we present this as the most supported model. This model retains the amount of forest land owned, age, technical knowledge level and PBC as predictors of xaté cultivation. Figure 4 presents the coefficient estimates for the most supported model. This illustrates that older farmers with technical knowledge about xaté cultivation and positive perceived behavioural control are the most likely to cultivate xaté.

**Figure 5:**
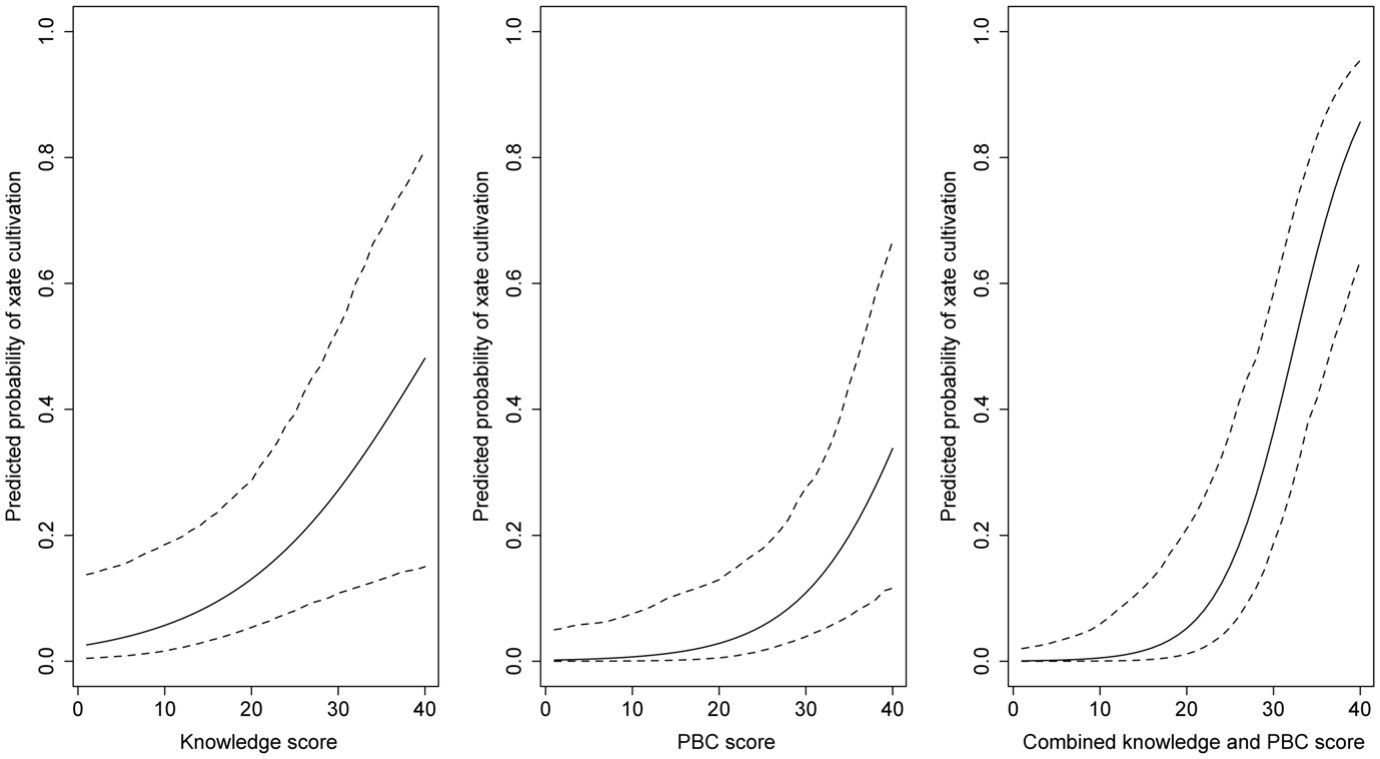
Influence of changing technical knowledge and perceived behavioural control on predicted probability of xaté cultivation. 5a Technical knowledge scores, 5b Perceived behavioural control (PBC) scores and 5c combined technical knowledge and perceived behavioural control score. Solid lines are the mean estimate and dashed lines are the 95% confidence intervals. Other parameters (age, forest ownership) were held at their median values whilst the parameter of interest was varied.

We used the best model to predict the impact of these two predictors on the probability of cultivating xaté. Figure 5 presents the simulations of different levels of technical knowledge (5a) and perceived behavioural control (5b). These illustrate that even with the highest level of knowledge or perceived behavioural control, the probability of cultivating xaté is less than 50%. However, when both perceived behavioural control and knowledge are increased to the highest levels simultaneously, the probability of cultivation rises to over 80% (figure 5c).

### Additional barriers to cultivation

Farmers reported two additional perceived barriers to xaté cultivation through the semi-structured interviews: the lack of market to sell the leaves in Belize (quote 1) and access to xaté seeds (quote 2).

*There is an export market in Guatemala, but in Belize we don't have it. I don't know why there is no market here -* Not xaté farmer, not trained [quote 1]

*We don't have the seed, we don't have the financial support or government support to start xaté. I don't think there is a company in Belize to buy seed. I think to get seed we will have to go to the jungle –* Not xaté farmer, trained [quote 2]

In addition to the two reported barriers, farmers in the village closest to the Guatemalan border stated that theft was a problem when cultivating xaté. It was generally believed that Guatemalans illegally crossing the border carry out these occurrences of theft (quotes 3 and 4).

*‘Guatemalans are entering our country and they go into our land. They cut the xaté and they take it. When we are not there, they take it.’* – Previously a xaté farmer, trained [quote 3]

*They come in from Guatemala and come in Belize, take our xaté from the forest. In the future we will have nothing to show what is xaté* – Xaté farmer, trained [quote 4]

## Discussion

Training programmes are widely used to influence behaviour and have been found to be useful in a range of fields, including improving driving behaviour [38], improving health worker practices [39] and increasing the use of malaria prevention techniques [40]. By targeting the factors known to influence behaviour, training programmes can potentially encourage the adoption of new behaviours. Here we discuss the impact of a cultivation training programme on the four proposed predictors of behaviour, and the impact of training on behaviour. We recognise that a limitation of this study is that participants in the initial training programme were self-selecting and differences between the trained and untrained group may have existed before the training programme. We found no difference in the measured socio-demographic characteristics between the trained and untrained groups, suggesting the differences may be relatively small. However, without random assignment of participants to the training programme a possible bias is unavoidable it is therefore not possible with complete confidence to conclude that the training programme caused the differences observed.

### The effect of training

We found training had a small positive impact on attitudes and no evidence of influence upon subjective norms, in the context of xaté cultivation. Before the training programme the Belize government had been promoting xaté cultivation [41], which may have resulted in universally positive attitudes and subjective norms among farmers in the study area. Alternatively, training may have influenced attitudes and subjective norms of the participants and these perceptions were then, over time, transferred to other farmers in the area. Such peer to peer transfer of information is commonly a stated objective of agricultural development interventions [42]–[43] and could explain the small difference in the attitudes and subjective norms of trained and untrained informants. There is a strong contrast between this lack of difference and the clear difference between trained and untrained individuals in their technical knowledge and perceived behavioural control. It is remarkable that this division has been maintained in the 5 years since the training course. This finding suggests that training did have a positive influence on technical knowledge and positive beliefs about the ability to cultivate xaté but this did not diffuse through the community. Previous studies have demonstrated training programmes can result in a transfer of knowledge within a community [44]–[45]. However, these studies focus on general knowledge about environmental issues whereas our study examines technical knowledge, which may be more difficult to transfer. Despite the lack of transfer of knowledge through the community, it is encouraging to find our results show training can influence technical knowledge and this knowledge is retained over five years. As perceived behavioural control was also increased, we assume the training programme addressed both of the facets of perceived behavioural control: training has influenced an individual's self belief in their ability, perhaps indirectly influenced through increasing technical knowledge. Secondly, to increase perceived behavioural control, training programmes need to address the resource access to help individuals implement a behaviour [30].

### Requirements for cultivation

A lack of knowledge in silvicultural and agroforestry practices is thought to hinder adoption of cultivation practices [46] and high confidence is thought to increase the intention to cultivate a novel species [24]. The predicted simulations highlight the importance of technical knowledge and perceived behavioural control as predictors of cultivation behaviour. Simultaneously increasing both knowledge and perceived behavioural control substantially increases the probability an individual will cultivate xaté. Training programmes are more likely to result in behavioural change if they can address the technical knowledge needed to cultivate a new species and also generate self confidence in individuals with the provision of resources needed to initiate cultivation. By providing seedlings to participants, the Belize Botanic Garden addressed the need for resources to establish xaté plantations. However, even if prospective cultivators have technical knowledge, self confidence in their abilities and seedlings, additional constraints may restrict implementation of cultivation [46]. There may be barriers to cultivation that training programmes are not able to address. We identify three factors that people perceive as important considerations before initiating xaté cultivation: access to seeds, lack of market and theft of xaté. There are no xaté nurseries selling seedlings or seed and our study found evidence of seed harvesting from wild populations. It has been suggested that harvest of palm seeds can be more detrimental than harvesting leaves [47]. We therefore suggest caution should be taken when promoting cultivation of a new species if there is no sustainable source of seeds or seedlings after the training programme has been completed. The lack of an established xaté market in Belize discourages farmers from investing in xaté cultivation. Although it may not be the responsibility of the training coordinators to establish the market, it is perhaps important for the organisation to consider this important factor before encouraging individuals to cultivate a new crop. With high value plant products, theft is a potential risk for farmers. Our study shows how this risk can be high enough to deter farmers from cultivating a new species such as xaté. Training programmes are unlikely to alter behaviour if theft of the plant is likely and this needs to be considered before expecting individuals to invest in cultivation of a new species.

### Conclusion

The theory of planned behaviour has provided a useful framework for examining factors that can predict behaviour. Our study illustrates how training programmes can influence behaviour and how this can encourage cultivation of over-harvested plant species. We show that training programmes can influence participants' technical knowledge and their self assessment of whether a behaviour can be enacted successfully (perceived behavioural control) and that these variables are important in predicting whether people take up cultivation. It is interesting to note that technical knowledge and perceived behavioural control did not appear to transfer between participants and non-participants over the five year period between the training programme and our research. Future training programmes aimed at increasing cultivation of over-harvested plants therefore need to target both individuals' technical knowledge and perceived behavioural control, which may be influenced by providing the seeds or seedlings needed for cultivation. Whether cultivation is an effective approach for reducing pressure on wild populations still requires further research.

## Supporting Information

Supplementary Material S1 **Ethics Statement.**
(PDF)
