# Supplementary material for: Training Programmes Can Change Behaviour and Encourage the Cultivation of Over-Harvested Plant Species
Source: PLoS One. 2012 Mar 14;7(3):e33012. doi: 10.1371/journal.pone.0033012 (PMC3303790; doi:10.1371/journal.pone.0033012)
Supplement: Supplementary Material S1 — Ethics Statement. (PDF) [file pone.0033012.s001.pdf]

COLEG Y GWYDDORAU NATURIOL
COLLEGE OF NATURAL SCIENCES

PRIFYSGOL
BANGOR
UNIVERSITY

Dr Julia Jones
School of the Environment, Natural Resources and Geography

1 September 2011

Dear Julia

I am writing to confirm that the screening review of your proposed research on *'Training programmes can change behaviour and encourage the cultivation of over-harvested plant species'* indicated that it did not require further consideration by the College of Natural Sciences Ethics Committee.

This is because the research does not involve:

- NHS patients, relatives or carers
- children or vulnerable adults
- live vertebrates or *Octopus vulgaris*
- fieldwork where permission from landowners or other authorities is required
- use of a gatekeeper for access to groups or individuals
- deception or conduction without participants' full and informed consent
- discussion of sensitive topics
- intrusive interventions
- psychological stress, anxiety or humiliation
- personal or confidential information
- collection or storage of human tissues

Yours sincerely

Michelle Jones

Michelle Jones
Secretary, College of Natural Sciences Ethics Committee

COLEG Y GWYDDORAU NATURIOL
PRIFYSGOL BANGOR
ADEILAD MEMORIAL
FFORDD DEINIOL
BANGOR, GWYNEDD
LL57 2UW

FFÔN: +44 (01248) 382603
FFACS: +44 (01248) 370731

WWW.BANGOR.AC.UK

COLLEGE OF NATURAL SCIENCES
BANGOR UNIVERSITY
MEMORIAL BUILDING
DEINIOL ROAD
BANGOR, GWYNEDD
LL57 2UW, UK

TEL: +44 (01248) 382603
FAX: +44 (01248) 370731

WWW.BANGOR.AC.UK/CNS
